# Noise annoyance due to different sources is associated with tinnitus presence and distress in the general population

**DOI:** 10.1038/s41370-024-00668-9

**Published:** 2024-04-03

**Authors:** Omar Hahad, Julia Döge, Katharina Bahr-Hamm, Manfred E. Beutel, Konstantin Kontohow-Beckers, Alexander K. Schuster, Karsten Keller, Lukas Hobohm, Volker H. Schmitt, Emilio Gianicolo, Karl J. Lackner, Andreas Daiber, Philipp S. Wild, Berit Hackenberg, Thomas Münzel

**Affiliations:** 1 Department of Cardiology – Cardiology I, University Medical Center of the Johannes Gutenberg-University Mainz, Mainz, Germany; 2 German Center for Cardiovascular Research (DZHK), partner site Rhine-Main, Mainz, Germany; 3 Department of Otorhinolaryngology, University Medical Center of the Johannes Gutenberg-University Mainz, Mainz, Germany; 4 Department of Psychosomatic Medicine and Psychotherapy, University Medical Center of the Johannes Gutenberg-University Mainz, Mainz, Germany; 5 Preventive Cardiology and Preventive Medicine, Department of Cardiology, University Medical Center of the Johannes Gutenberg-University Mainz, Mainz, Germany; 6 Department of Ophthalmology, University Medical Center of the Johannes Gutenberg-University Mainz, Mainz, Germany; 7 Center for Thrombosis and Hemostasis (CTH), University Medical Center of the Johannes Gutenberg-University Mainz, Mainz, Germany; 8 Medical Clinic VII, Department of Sports Medicine, University Hospital Heidelberg, Heidelberg, Germany; 9 Institute of Medical Biostatistics, Epidemiology & Informatics, University Medical Center of the Johannes Gutenberg-University Mainz, Mainz, Germany; 10 Institute of Clinical Physiology, National Research Council, Lecce, Italy; 11 Institute of Clinical Chemistry and Laboratory Medicine, University Medical Center of the Johannes Gutenberg-University Mainz, Mainz, Germany; 12 Institute for Molecular Biology, Mainz, Germany

**Keywords:** Noise annoyance, Stress, Environmental risk factor, Tinnitus, Tinnitus distress, Auditory system

## Abstract

**Background:**

The pathophysiology of tinnitus is not yet fully understood. Although there is a large amount of evidence associating traffic noise exposure with non-auditory health outcomes, there is no evidence regarding the impact of noise annoyance on auditory disorders such as tinnitus.

**Objective:**

Thus, we aimed to investigate the association between noise annoyance due to different sources and tinnitus presence and distress in the general population.

**Methods:**

Data of 6813 participants from a large German population-based cohort were used (Gutenberg Health Study). Participants were asked about the presence of tinnitus and how much they were bothered by it. In addition, information on annoyance from road traffic, aircraft, railways, industrial, and neighborhood noise during the day and sleep was collected through validated questionnaires.

**Results:**

The prevalence of tinnitus was 27.3%, and the predominant sources of noise annoyance in these subjects were aircraft, neighborhood, and road traffic noise. Overall, logistic regression results demonstrated consistent positive associations between annoyance due to different noise sources and prevalent risk of tinnitus with increases in odds ratios ranging from 4 to 11% after adjustment for sex, age, and socioeconomic status. Likewise, consistent increases in odds ratios were observed for tinnitus distress in subjects with prevalent tinnitus. For instance, neighborhood noise annoyance during the sleep was associated with a 26% increase in tinnitus distress (OR 1.26, 95% CI 1.13; 1.39).

**Impact:**

This is the first study investigating the association between noise annoyance and tinnitus presence and distress in a large cohort of the general population. Our results indicate consistent and positive associations between various sources of noise annoyance and tinnitus. These unprecedented findings are highly relevant as noise annoyance and tinnitus are widespread. The precise etiology and locus of tinnitus remain unknown, but excessive noise exposure is thought to be among the major causes. This study suggests that transportation and neighborhood noise levels thought merely to contribute to annoyance and non-auditory health effects may be sufficient to cause or exacerbate tinnitus.

## Introduction

The term tinnitus is defined as the conscious perception of a sound in the absence of a stimulus from an external acoustic source [1]. The pathophysiology of tinnitus is complex and multifactorial [2, 3]. Tinnitus, arising from pathological changes in the auditory pathway, often develops due to initial cochlear lesions like sudden hearing loss, noise trauma, presbyacusis, or ototoxic drug use. The association between hearing loss and tinnitus is intricate, with not all hearing-impaired individuals experiencing tinnitus, and abnormal audiograms not universally present in those with tinnitus. Additionally, partial cochlear nerve sections may not impact hearing thresholds, and tinnitus in those with normal hearing may be linked to cochlear dead regions or outer hair cell damage. Tinnitus onset can be influenced by temporomandibular joint disorders, neck injuries, emotional stress, and trauma. The multifactorial nature of tinnitus involves a combination of altered auditory and somatosensory inputs, abnormal central nervous system activity, and emotional factors, particularly evident in tinnitus following traumatic head injuries. The factors contributing to tinnitus generation may differ from those related to its persistence, as seen in cases of transient tinnitus after noise trauma [2, 3]. Exacerbating factors related to the transition from acute to chronic tinnitus may include mental stress, other psychological factors, and tinnitus-related distress [4, 5]. Interestingly, the hypothesis was put forward that stress per se may cause tinnitus [6]. In particular, it was demonstrated that stressful situations and sleep disturbances precede tinnitus onset and progression from mild to bothersome symptoms [7–9]. As stress and sleep disturbance are proposed key mechanisms behind the harmful health effects of environmental noise exposure [10, 11], the hypothesis was formulated that exposure to transportation noise levels—typically below the thresholds associated with hearing damage [12]—can influence the onset and discomfort caused by tinnitus. Indeed, a recent nationwide cohort study from Denmark demonstrated that residential exposure to road traffic noise may increase risk of incident tinnitus [13]. However, it remains to be established whether noise annoyance, which is regarded as an important effect modifier of the relation between noise exposure and non-auditory health outcomes including cardiovascular disease [14], is associated with tinnitus.

Noise annoyance may be an interesting indicator to investigate in this setting. Firstly, noise annoyance strongly correlates with mental stress and health outcomes such as depression and anxiety [15]. Secondly, noise annoyance is among the most significant effects in the general population caused by environmental noise exposure [10]. Moreover, the World Health Organization acknowledged in 2018 that studies investigating the association between transportation noise and hearing-related outcomes, such as tinnitus, are lacking [16].

To the best of our knowledge, there are no studies to date examining the association between noise annoyance and prevalent risk of tinnitus as well as tinnitus distress in the general population. Thus, we used data from a large population-based cohort associating noise annoyance due to different sources and tinnitus presence and distress.

## Methods

### Study design and sample

Data from the Gutenberg Health Study (GHS) were used for analysis. Comprehensive information on the study design and details were published previously [17, 18]. In brief, 15,010 individuals (aged 35–74 years, core cohort) underwent a standardized 5-h-long baseline-examination performed from 2007 to 2012 at the study center of the University Medical Center Mainz, Germany. These examinations included a variety of interviews and clinical examinations conducted in compliance with standard protocols. The follow-up examinations took place 5 and 10 years after enrollment, i.e., from 2012 to 2017 and from 2017 to 2022. At this 10-year follow-up (from 2017 to 2020), otologic testing was included in the study design. In addition to the core cohort, new participants aged 25–44 years (young cohort, *n* = 4000) and 75–85 years (senior cohort, *n* = 1000) were recruited. All three cohorts will be followed up in 10-year increments through 2027 [19].

All procedures conducted in the GHS were approved by the ethics committee of the Statutory Physician Board of the State Rhineland-Palatinate [reference number 837.020.07(5555)] and the local data safety commissioners and were in line with the ethical principles for medical research involving human subjects as outlined in the Declaration of Helsinki. Before inclusion in the study, written informed consent was obtained from each participant.

### Noise annoyance

Noise annoyance was measured in a standardized and validated manner as reported recently [20, 21] and endorsed by the International Commission on Biological Effects of Noise (ICBEN) in 2001 [22]. Using a five-point Likert scale with distinct semantic differentiations, approximately equidistant from each other, ranging from “not at all,” over “slightly,” “moderately,” and “strongly” to “extremely,” participants were asked to rate “how annoyed have you been in the past years by … during the day/in your sleep?”. Various sources of noise annoyance including road traffic, aircraft, railway, industrial, and neighborhood noise were assessed. The calculation of overall noise annoyance involved considering the highest annoyance rating reported by participants, irrespective of the specific noise source or whether the annoyance occurred during the day or in their sleep. Overall noise annoyance represents a comprehensive measure that captures the participants’ highest reported level of annoyance, encompassing various noise sources such as road traffic, aircraft, railway, industrial, and neighborhood noise. This calculation disregards the distinction between daytime and sleep-related annoyance, focusing on the intensity of the overall experience. Additionally, source-specific overall noise annoyance narrows down the focus to the specific noise source that contributed the most to an individual’s overall noise annoyance, regardless of its impact on daytime or sleep. This approach aims to provide a more detailed understanding of how different noise sources contribute to the participants’ overall perception of annoyance. Both overall noise annoyance and source-specific overall noise annoyance can be understood as continuous variables, given that they result from the 5-point Likert scale.

### Tinnitus presence and distress

During a standardized interview, participants were asked to complete a questionnaire about their auditory quality of life including standardized questions on tinnitus presence and distress [19]: “Do you suffer from ringing in the ears (tinnitus)?” with a binary answer format (yes/no) and “How much do you feel bothered by it?” with a six-level answer format and Likert scale ranging from 0 (“not stressful”) to 5 (“very stressful”).

### Statistical analysis

Participants with missing data on noise annoyance were excluded from the analysis. Differences in continuous study sample characteristics, such as age and socioeconomic status, were assessed using the t-test. For dichotomous variables, such as sex, the chi-square test was employed. Binary logistic regression analysis with corresponding odds ratios (OR), 95% CI, and *P* values were used to determine the relationship between noise annoyance and prevalent tinnitus. Noise annoyance was treated as a continuous variable (per point increase) in all models. In those participants with prevalent tinnitus, ordinal logistic regression analysis with proportional odds assumption modeling for tinnitus distress was performed. Statistical analysis included sequential adjustment. Model 1 was adjusted for sex (binary). Model 2 was additionally adjusted for age (continuous). Model 3 was additionally adjusted for socioeconomic status (continuous). The socioeconomic status was assessed by a validated index score (ranging from 3 (lowest) to 21 (highest)), providing information about education, occupation, and household net-income [23, 24]. In the present analysis, *P*-values should be interpreted as a continuous measure of evidence against the null hypothesis, and they are therefore reported exactly. For descriptive reasons, *P*-values < 0.05 were regarded as important associations. The statistical data analyses were performed using the software R (http://www.r-project.org/).

## Results

### Study sample characteristics

After excluding participants without information on noise annoyance, a total of *N* = 6,813 participants were included in the present analysis. Among these, 1,862 (27.3%) suffered from tinnitus. In participants with prevalent tinnitus, more than the half reported no (rating 0–24.7%) or little (rating 1-34.8%) suffering from tinnitus. Participants with prevalent tinnitus were more likely to be men (56.5%, *P* < 0.0001), were older (mean age 58.0 ± 10.8 vs. 55.8 ± 11.8, *P* < 0.0001), and had a slightly lower socioeconomic status (14.33 ± 4.18 vs. 14.46 ± 4.15, *P* = 0.27). Overall, annoyance due to aircraft (63.6%), neighborhood (46.8%), and road traffic (46.3%) noise were most frequent in participants with prevalent tinnitus.

### Association between noise annoyance and prevalent tinnitus

Tables 1 and 2 show the results of the logistic regression analysis concerning the association between noise annoyance and prevalent tinnitus. In general, consistent positive associations between annoyance due to different noise sources and prevalent risk of tinnitus were observed. Increases in ORs ranged from 4 to 11% after multivariable adjustment with industrial noise annoyance during the day displaying an OR of 1.11 (95% CI 1.03; 1.19).

**Table 1:** Odds ratio (OR) and 95% confidence intervals (CIs) derived from three binary logistic regression models modeling for prevalent tinnitus (dependent variable) per point increase in (source-specific) overall noise annoyance (independent variable).

(Source-specific) Overall noise annoyance	*N*	Model 1		Model 2		Model 3	
		OR per point increase [95% CI]	*P*-value	OR per point increase [95% CI]	*P*-value	OR per point increase [95% CI]	*P*-value
Road traffic	6,603	1.06 [1.01; 1.12]	**0.028**	1.06 [1.01; 1.12]	**0.027**	1.07 [1.01; 1.13]	**0.015**
Aircraft	6,603	1.08 [1.04; 1.13]	**0.00026**	1.07 [1.02; 1.11]	**0.0021**	1.07 [1.03; 1.12]	**0.0012**
Railway	6,603	1.07 [0.99; 1.16]	0.070	1.08 [1.00; 1.16]	0.062	1.07 [0.99; 1.16]	0.070
Industrial	6,603	1.08 [1.00; 1.15]	**0.042**	1.10 [1.02; 1.18]	**0.011**	1.10 [1.02; 1.18]	**0.0090**
Neighborhood	6,603	1.03 [0.97; 1.09]	0.28	1.07 [1.01; 1.14]	**0.016**	1.08 [1.02; 1.14]	**0.011**
Overall	6,603	1.06 [1.02; 1.11]	**0.0069**	1.06 [1.02; 1.11]	**0.0052**	1.07 [1.02; 1.11]	**0.0038**

*N* denotes model 3. Source-specific overall noise annoyance was defined as highest source-specific annoyance rating regardless of whether it affected daytime or sleep. Overall noise annoyance was defined as highest annoyance rating regardless of the specific noise source and of whether it affected daytime or sleep.

Model 1 was adjusted for sex.

Model 2 was additionally adjusted for age.

Model 3 was additionally adjusted for socioeconomic status.

*P*-values < 0.05 (in bold) were regarded as important associations.

**Table 2:** Odds ratio (OR) and 95% confidence intervals (CIs) derived from three binary logistic regression models modeling for prevalent tinnitus (dependent variable) per point increase in noise annoyance during the day and sleep (independent variable).

Noise annoyance	*N*	Model 1		Model 2		Model 3	
		OR per point increase [95% CI]	*P*-value	OR per point increase [95% CI]	*P*-value	OR per point increase [95% CI]	*P*-value
*Day*							
Road traffic	6,602	1.06 [1.00; 1.13]	**0.033**	1.06 [1.00; 1.13]	**0.038**	1.07 [1.01; 1.13]	**0.024**
Aircraft	6,603	1.09 [1.04; 1.14]	**0.00013**	1.07 [1.03; 1.12]	**0.0012**	1.08 [1.03; 1.13]	**0.00091**
Railway	6,600	1.06 [0.98; 1.15]	0.16	1.06 [0.98; 1.15]	0.15	1.06 [0.98; 1.15]	0.16
Industrial	6,601	1.08 [1.01; 1.16]	**0.031**	1.10 [1.02; 1.18]	**0.0087**	1.11 [1.03; 1.19]	**0.0061**
Neighborhood	6,603	1.04 [0.98; 1.10]	0.23	1.08 [1.02; 1.15]	**0.012**	1.09 [1.02; 1.16]	**0.0079**
Overall	6,603	1.07 [1.03; 1.12]	**0.0021**	1.07 [1.03; 1.12]	**0.0018**	1.07 [1.03; 1.12]	**0.0014**
*Sleep*							
Road traffic	6,590	1.07 [0.99; 1.15]	0.10	1.08 [1.00; 1.17]	**0.047**	1.09 [1.01; 1.18]	**0.027**
Aircraft	6,590	1.04 [1.00; 1.09]	0.075	1.03 [0.99; 1.08]	0.18	1.04 [0.99; 1.09]	0.15
Railway	6,590	1.09 [0.99; 1.20]	0.089	1.09 [0.99; 1.21]	0.074	1.09 [0.99; 1.21]	0.084
Industrial	6,590	1.02 [0.86; 1.19]	0.82	1.05 [0.89; 1.22]	0.57	1.06 [0.90; 1.24]	0.48
Neighborhood	6,590	1.04 [0.96; 1.12]	0.33	1.08 [1.00; 1.16]	**0.048**	1.09 [1.00; 1.17]	**0.036**
Overall	6,591	1.04 [0.99; 1.08]	0.083	1.04 [1.00; 1.09]	0.057	1.05 [1.00; 1.09]	**0.045**

*N* denotes model 3.

Model 1 was adjusted for sex.

Model 2 was additionally adjusted for age.

Model 3 was additionally adjusted for socioeconomic status.

*P*-values < 0.05 (in bold) were regarded as important associations.

### Association between noise annoyance and tinnitus distress

Tables 3 and 4 show the results of the logistic regression analysis regarding the association between noise annoyance and tinnitus distress in participants with prevalent tinnitus. Again, consistent positive associations between annoyance due to different noise sources and tinnitus distress were observed. Increases in ORs up to 26% after multivariable adjustment were observed. Herein, neighborhood noise annoyance during the sleep was associated with a 26% increase in tinnitus distress (OR 1.26, 95% CI 1.13; 1.39).

**Table 3:** Odds ratio (OR) and 95% confidence intervals (CIs) derived from three ordinal logistic regression models with proportional odds assumption modeling for tinnitus distress in subjects with prevalent tinnitus (dependent variable) per point increase in (source-specific) overall noise annoyance (independent variable).

(Source-specific) Overall noise annoyance	*N*	Model 1		Model 2		Model 3	
		OR per point increase [95% CI]	*P*-value	OR per point increase [95% CI]	*P*-value	OR per point increase [95% CI]	*P*-value
Road traffic	1839	1.18 [1.09; 1.29]	**<0.0001**	1.19 [1.09; 1.30]	**<0.0001**	1.18 [1.08; 1.28]	**0.00017**
Aircraft	1839	1.18 [1.11; 1.26]	**<0.0001**	1.17 [1.10; 1.25]	**<0.0001**	1.20 [1.12; 1.28]	**<0.0001**
Railway	1839	0.99 [0.88; 1.11]	0.86	1.00 [0.89; 1.12]	0.98	1.00 [0.89; 1.13]	0.97
Industrial	1839	1.18 [1.06; 1.32]	**0.00029**	1.22 [1.09; 1.37]	**0.00049**	1.23 [1.10; 1.39]	**0.00040**
Neighborhood	1839	1.14 [1.04; 1.25]	**0.0037**	1.18 [1.08; 1.29]	**0.00025**	1.19 [1.09; 1.30]	**0.00019**
Overall	1839	1.20 [1.12; 1.28]	**<0.0001**	1.21 [1.13; 1.29]	**<0.0001**	1.22 [1.14; 1.31]	**<0.0001**

*N* denotes model 3. Source-specific overall noise annoyance was defined as highest source-specific annoyance rating regardless of whether it affected daytime or sleep. Overall noise annoyance was defined as highest annoyance rating regardless of the specific noise source and of whether it affected daytime or sleep.

Model 1 was adjusted for sex.

Model 2 was additionally adjusted for age.

Model 3 was additionally adjusted for socioeconomic status.

*P*-values < 0.05 (in bold) were regarded as important associations.

**Table 4:** Odds ratio (OR) and 95% confidence intervals (CIs) derived from three ordinal logistic regression models with proportional odds assumption modeling for tinnitus distress in subjects with prevalent tinnitus (dependent variable) per point increase in noise annoyance during the day and sleep (independent variable).

Noise annoyance	*N*	Model 1		Model 2		Model 3	
		OR per point increase [95% CI]	*P*-value	OR per point increase [95% CI]	*P*-value	OR per point increase [95% CI]	*P*-value
*Day*							
Road traffic	1838	1.18 [1.08; 1.29]	**0.00016**	1.19 [1.09; 1.30]	**0.00011**	1.17 [1.07; 1.28]	**0.00045**
Aircraft	1839	1.20 [1.12; 1.28]	**<0.0001**	1.19 [1.11; 1.27]	**<0.0001**	1.21 [1.13; 1.29]	**<0.0001**
Railway	1838	1.01 [0.89; 1.14]	0.92	1.02 [0.90; 1.15]	0.80	1.02 [0.90; 1.16]	0.76
Industrial	1839	1.19 [1.06; 1.33]	**0.00026**	1.23 [1.09; 1.37]	**0.00046**	1.23 [1.10; 1.39]	**0.00045**
Neighborhood	1839	1.09 [0.99; 1.19]	0.077	1.13 [1.03; 1.24]	**0.011**	1.14 [1.03; 1.25]	**0.0077**
Overall	1839	1.20 [1.12; 1.29]	**<0.0001**	1.21 [1.13; 1.30]	**<0.0001**	1.22 [1.14; 1.31]	**<0.0001**
*Sleep*							
Road traffic	1833	1.14 [1.01; 1.29]	**0.028**	1.16 [1.03; 1.31]	**0.016**	1.14 [1.01; 1.29]	**0.030**
Aircraft	1832	1.09 [1.02; 1.17]	**0.015**	1.08 [1.01; 1.16]	**0.024**	1.11 [1.04; 1.20]	**0.0033**
Railway	1832	1.00 [0.86; 1.17]	0.96	1.02 [0.88; 1.18]	0.80	1.03 [0.88; 1.20]	0.70
Industrial	1832	1.06 [0.83; 1.36]	0.65	1.08 [0.84; 1.38]	0.55	1.07 [0.83; 1.38]	0.58
Neighborhood	1832	1.21 [1.07; 1.36]	**0.0020**	1.24 [1.10; 1.40]	**0.00036**	1.26 [1.11; 1.42]	**0.00022**
Overall	1833	1.11 [1.04; 1.19]	**0.0022**	1.11 [1.04; 1.19]	**0.0014**	1.14 [1.07; 1.22]	**0.00014**

Odds ratios (OR) and 95% confidence intervals (CI) are derived from an ordinal logistic regression model with proportional odds assumption modeling for tinnitus distress in subjects with prevalent tinnitus (dependent variable) per point increase in noise annoyance (independent variable). *N* denotes model 3.

Model 1 was adjusted for sex.

Model 2 was additionally adjusted for age.

Model 3 was additionally adjusted for socioeconomic status.

## Discussion

To the best of our knowledge, this is the first study that observed an association between noise annoyance due to different sources and tinnitus presence and distress in a large cohort of the general population. Notably, the prevalence of tinnitus was 27.3%, being a representative estimate when comparing with previous estimates indicating a prevalence range from 9 to 28% in Europe [25]. Overall, we observed consistent and positive OR increases for the association between noise annoyance and prevalent tinnitus, which were robust to adjustment for sex, age, and socioeconomic status. No substantial OR differences between noise annoyance during the day and sleep were observed. Likewise, there was a consistent and positive association between noise annoyance and tinnitus distress in participants with prevalent tinnitus after multivariable adjustment. Our results strongly show that noise annoyance can be a reliable indicator of tinnitus presence and distress. Importantly, future studies have to investigate whether noise annoyance can also predict the onset of tinnitus.

Our results are in accordance with a recently published study from Cantuaria et al. that found a positive association between noise exposure and incident tinnitus [13]. In this nationwide cohort study including all residents from Denmark aged ≥ 30 years (40,692 were diagnosed with tinnitus at follow-up), the authors observed an increased hazard ratio of 1.06 (95% CI 1.04; 1.08) per 10 dB increase in road traffic noise exposure. Consequently, the authors concluded that road traffic noise exposure can negatively affect the auditory system. However, it is well-established that environmental noise exposure acts as a psychosocial stressor. According to the noise reaction scheme developed by Babisch [26], exposure to excessive noise levels can via an direct pathway induce hearing organ damage and sleep disturbance leading to neuroendocrine stress responses and thus to onset and progression of chronic non-communicable disease including cardiovascular disease [27, 28]. The indirect pathway refers to the exposure of lower noise levels, compatible with the exposure to traffic noise, interfering with daily activities, communication, and sleep, thus promoting stress responses and impairing health. Understanding the auditory system involves considering the pathway from the cochlea to the temporal cortex. Different points along this route could contribute to the perception of tinnitus. The primary impact of noise is typically on the outer hair cells in the cochlea, leading to noise-induced hearing loss. However, research suggests that noise exposure goes beyond causing damage to outer hair cells, it can also result in synaptopathy, affecting the synapses between inner hair cells and the spiral ganglion neuron. Thus, it is likely that a certain level of noise intensity, higher than typical environmental noise exposure levels, is necessary for these effects to occur. While environmental noise exposure is rather not likely to cause distinct cochlear damage, it is considered a potential factor in triggering tinnitus perception via the induction of neuroplastic changes in the central auditory system. The complex interplay between environmental noise, the structural integrity of the auditory system, and the intricacies of tinnitus perception highlights the multifaceted nature of the auditory response to noise exposure [2, 3]. Our results expand on the findings of Cantuaria et al. [13] by indicating that also non-traffic noise annoyance such as neighborhood and industrial noise annoyance associate with tinnitus presence as well as distress. These findings are in line with the evidence that stress may play a major role in the development, maintenance, and worsening of tinnitus [7, 29].

On a more mechanistic level, increased stress hormone levels may negatively affect the limbic, reticular, and auditory systems, thereby initiating or aggravating tinnitus [30]. An increase in stress hormone levels is likely accompanied by inflammatory and oxidative stress processes. A series of human and animal studies conducted by our research group demonstrated that traffic noise exposure could increase stress hormone levels, disturb sleep, and increase inflammation and oxidative stress, leading to vascular endothelial dysfunction (for overview see [27]). Indeed, there is evidence showing a close relationship between oxidative stress, nitric oxide, endothelial dysfunction, and tinnitus [31]. Study results from Neri et al. suggested that in acute tinnitus patients, levels of oxidative stress are increased and nitric oxide production is reduced being able to induce cerebrovascular endothelial dysfunction, which in turn induce a dysfunction of the microcirculation of the inner ear [31]. However, it is important to note that chronic tinnitus associated with long-term stress may not have a direct correlation with the microcirculation of the inner ear. The relationship between chronic stress and tinnitus likely involves intricate interactions across neural and psychological factors, necessitating further research for a comprehensive understanding [6, 9].

Disturbed sleep by noise exposure may also constitute an important underlying mechanism, as studies not only demonstrated impaired sleep in tinnitus patients but also sleep disturbance to precede tinnitus distress [32]. In support of this, we have recently demonstrated that noise annoyance can predict the onset of sleep disturbance in the GHS [15]. In this setting, a vicious cycle can be triggered in which sleep disturbance would worsen tinnitus symptoms and more bothersome symptoms would lead to impaired sleep [33]. In favor of this, tinnitus was shown to be louder and more distressing during the night and in the early morning [34]. Thus, it can be hypothesized that noise-induced stress and annoyance during the night along with fragmentation of sleep, may increase tinnitus patients’ awareness of tinnitus and subsequent level of distress when they try to resume sleep [13].

The present study has strengths and potential limitations. Strengths of the present study include the underlying large dataset from a large population-representative sample from Germany. Some important limitations, however, need to be considered. First, the observational and cross-sectional nature of the study does not allow for causal inferences. Noise annoyance and the outcomes of interest were assessed by personal interviews and partly validated questionnaires only that largely depend on the individual compliance. Specifically, we did not include the duration of tinnitus or differentiate between intermittent and continuous symptoms in our questionnaire. Unlike common definitions that specify sounds lasting for more than five minutes, our questionnaire did not set a minimum duration for tinnitus symptoms. These factors may influence the prevalence and characterization of tinnitus in our study, potentially affecting the comparability of our findings with research that uses different tinnitus definitions. However, diagnosing tinnitus is complicated as there is no objective test or a single organic correlate to confirm the occurrence of the disorder. Furthermore, there is likely a bidirectional relationship between noise annoyance and tinnitus (distress), which we did not analyze. However, it is important to note that evidence of a bidirectional association does not necessarily negate causal explanations in one or both directions of an association.

To the best of our knowledge, this is the first study investigating the association between noise annoyance and tinnitus presence and distress in a large cohort of the general population. Our results indicate consistent and positive associations between various sources of noise annoyance and the outcomes of interest. Potential mechanisms underlying these relationships refer to the noise reaction scheme. However, large prospective studies providing deep mechanistic insight are needed to confirm the proposed mechanisms.

## Data Availability

The analysis presents clinical data of a large-scale population-based cohort with ongoing follow-up examinations. This project constitutes a major scientific effort with high methodological standards and detailed guidelines for analysis and publication to ensure scientific analyses on the highest level. Therefore, data are not made available for the scientific community outside the established and controlled workflows and algorithms. To meet the general idea of verification and reproducibility of scientific findings, we offer access to data at the local database in accordance with the ethics vote on request at any time. The GHS steering committee, which comprises a member of each involved department and the head of the GHS, convenes once a month. The steering committee decides on internal and external access of researchers and use of the data and biomaterials based on a research proposal to be supplied by the researcher. Interested researchers make their requests to the head of the GHS (Philipp S. Wild, philipp.wild@unimedizin-mainz.de).
